# Synergistic Integration of Laboratory and Numerical Approaches in Studies of the Biomechanics of Diseased Red Blood Cells

**DOI:** 10.3390/bios8030076

**Published:** 2018-08-10

**Authors:** He Li, Dimitrios P. Papageorgiou, Hung-Yu Chang, Lu Lu, Jun Yang, Yixiang Deng

**Affiliations:** 1 Division of Applied Mathematics, Brown University, Providence, RI 02912, USA; lu_lu_1@brown.edu (L.L.); yixiang_deng@brown.edu (Y.D.); 2 Department of Materials Science and Engineering, Massachusetts Institute of Technology, Cambridge, MA 02139, USA; dpapag@mit.edu (D.P.P.); junyang@mit.edu (J.Y.); 3 School of Engineering, Brown University, Providence, RI 02912, USA

**Keywords:** red blood cell disorders, numerical modeling, laboratory approaches

## Abstract

In red blood cell (RBC) disorders, such as sickle cell disease, hereditary spherocytosis, and diabetes, alterations to the size and shape of RBCs due to either mutations of RBC proteins or changes to the extracellular environment, lead to compromised cell deformability, impaired cell stability, and increased propensity to aggregate. Numerous laboratory approaches have been implemented to elucidate the pathogenesis of RBC disorders. Concurrently, computational RBC models have been developed to simulate the dynamics of RBCs under physiological and pathological conditions. In this work, we review recent laboratory and computational studies of disordered RBCs. Distinguished from previous reviews, we emphasize how experimental techniques and computational modeling can be synergically integrated to improve the understanding of the pathophysiology of hematological disorders.

## 1. Introduction

Human red blood cells (RBCs) are nucleus-free cells, and they are comprised primarily of a concentrated solution of hemoglobin and an oxygen-binding protein, surrounded by a cell membrane [1]. The RBC membrane consists of two components: the lipid bilayer and the cytoskeleton. The lipid bilayer is approximately 5 nm thick and it is constituted mostly by a wide variety of lipids and protein molecules [2]. A typical lipid molecule consists of a hydrophilic polar head and two hydrophobic hydrocarbon chains. When introduced into an aqueous environment, the lipid molecules spontaneously aggregate into bilayer structure due to the hydrophobic effect. The RBC membrane cytoskeleton consists of spectrin tetramers, which are connected at the actin junctional complexes, forming a 2D sixfold structure. Spectrin is a protein tetramer formed by head-to-head association of two identical heterodimers [3]. Each heterodimer consists of an α-chain with 22 triple-helical segments and a β-chain with 17 triple-helical segments. The cytoskeleton is connected to the lipid bilayer via “immobile” band-3 proteins at the spectrin-ankyrin binding sites and via glycophorin proteins at the actin junctional complexes.

RBCs deliver oxygen from the lungs to the peripheral tissues, transporting carbon dioxide from these tissues back to the lungs for expiration. When circulating through the body, RBCs undergo repeated deformations as they pass through narrow pathways within the microvasculature. The remarkable deformability of RBCs results from the cell morphology and stiffness of the membrane cytoskeleton [4]. The innate biconcave shape of RBCs results in a high surface area-to-volume (S/V) ratio, contributing to their deformability. In addition, the membrane cytoskeleton, which endows RBCs with shear elasticity, exhibits a non-linear shear response under external shear forces, allowing RBCs to restore their biconcave shape after severe deformations [5]. The cohesion between the lipid bilayer and cytoskeleton maintains the stability of RBCs, preventing the surface area loss of RBCs in the circulation [6,7,8].

In RBC disorders, alterations to the morphology and membrane structure of RBCs lead to changes in their biomechanical properties. Sickle cell disease (SCD), an inherited RBC disorder, originates from the mutation of normal hemoglobin (HbA) to sickle hemoglobin (HbS) [9,10]. Sickle red blood cells (SS-RBCs) contain the mutant sickle hemoglobin (HbS), which polymerizes when subject to deoxygenation and eventually causes the cells to sickle. Sickling of RBCs induces a drastic dynamic alteration of the shape, the surface properties, and the overall cell rigidity of RBCs. Sickled RBCs can be visually identified via microscopy due to the severely distorted heterogeneous shapes under hypoxia compared to their respective shapes under normoxia, including elongated, granular, oval, holly-leaf, and crescent (classic sickle) shapes [11,12,13]. SS-RBCs have increased stiffness and are prone to adhesion, contributing to the initiation and propagation of vaso-occlusion events, a hallmark of SCD [14,15]. Recurrent and unpredictable episodes of vaso-occlusion in SCD lead to morbidity and reduced quality of life as a result of stroke and frequent painful crisis events [14,16,17,18,19,20].

Hereditary spherocytosis (HS), another inherited blood disorder, is caused by defects in the RBC membrane proteins that are responsible for the cohesion between cytoskeleton and lipid bilayer [21,22,23,24,25]. Reduced cohesion between these two layers can trigger loss of the membrane surface area through vesiculation [21]. Loss of the membrane surface results in the morphological changes of RBCs and thus compromises the cell deformability. As a result, these altered RBCs are removed prematurely by the spleen [26], leading to hemolytic anemia.

Diabetes mellitus (DM), a metabolic dysfunction, is induced by defects in the secretion and action of insulin, a hormone that regulates the blood sugar level in the human body. Type 2 DM (T2DM) is the most common form of diabetes, accounting for 90–95% cases of diabetic patients [27]. Diabetic patients are characterized as having abnormal hemorheology which is partially induced by the impaired deformability of RBCs. Diabetic RBCs are larger and stiffer than those of healthy subjects [28,29]. These abnormal RBCs cause increased blood viscosity and an insufficient blood supply, resulting in diabetic microangiopathy and other circulation problems [29,30].

Various laboratory approaches, such as atomic microscopy [31,32,33,34], optical tweezer [35,36,37,38], micropipette ascription [39,40,41,42], flow cytometry [43,44,45], ektacytometry [46,47,48,49,50] and numerous microfluidics studies (see summary in Table 1), have been developed to measure the biomechanical properties of diseased RBCs (see recent reviews [51,52,53]). Concurrently, computational RBC models, based on either particle methods [54,55,56,57,58,59,60,61,62,63] or continuum methods which implemented boundary integral method or immersed boundary method to couple RBC models with finite volume method, finite element method and the Lattice Boltzmann Method [64,65,66,67,68], have been constructed to probe RBC mechanics under physiological and pathological conditions (see recent reviews [69,70,71,72,73]). In the following text, we review the recent experimental findings and the corresponding numerical studies in SCD, HS, and T2DM and elaborate on how various technologies can be synergically integrated to improve the understanding of the pathophysiology of hematological disorders.

## 2. Sickle Cell Disease

### 2.1. Sickle Hemoglobin Fibers

**Figure 1:**
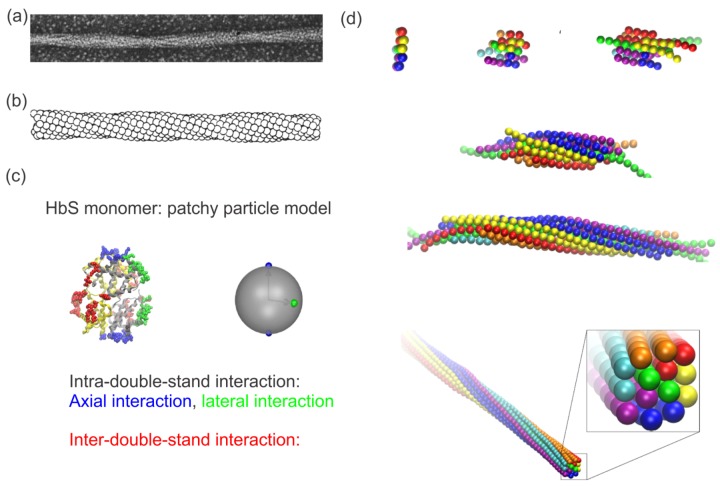
(**a**) Electronic microscopy image of structure of a single sickle hemoglobin (HbS) fiber. Reproduced with permission from reference [74]. (**b**) Reconstruction of the HbS fiber with a sphere model. Reproduced with permission from reference [74]. (**c**) Mesoscopic modeling of HbS molecules (**left**) by patchy particles (**right**). Green and blue represent lateral and axial intra-double-strand contacts. Red signifies the inter-double-strand contacts. Reproduced with permission from reference [81]. (**d**) Sequential snapshots of HbS polymerization from a nucleus to a fiber. Reproduced with permission from reference [81].

Hemoglobin is a group of oxygen-binding proteins inside RBCs. HbA consists of four connected globin subunits—two α and two β-globin subunits. In the mutated form of hemoglobin, HbS, valine replaces glutamate at the sixth position on the surface of the β-subunit, potentially causing polymerization of HbS molecules into fibers under deoxygenation. Both electron microscopy and X-ray diffraction have revealed that a single HbS fiber consists of seven double strands in a hexagonally-shaped cross-section twisted about a common axis in a rope-like fashion [74,75,76]. As shown in Figure 1a, the electron microscopy image of a single HbS fiber was obtained by recording samples negatively stained with 2% phosphotungstate using a Philips EM 301 electron microscope [74]. A Syntex AD-1 autodensitometer was connected to a Nova computer to perform Fourier transforms of the HbS fiber for fiber reconstructions, as shown in Figure 1b. The average diameter of a single HbS fiber was evaluated to be approximately 22 nm. Subsequently, based on the fiber structure identified by X-ray diffraction and three-dimensional reconstructions of electron microscopic images, three types of interactions were discovered within HbS fibers [77,78,79,80], namely (i) intra-double-strand axial interactions (highlighted in blue color in Figure 1c); (ii) intra-double-strand lateral interactions (highlighted in green color in Figure 1c); and (iii) inter-double-strand interactions (highlighted in red color in Figure 1c). Guided by these former findings, Lu et al. developed a coarse-grained (CG) patchy particle model for the HbS molecules [81]. As shown in Figure 1c (right), a HbS molecule in this model is represented by a spherical particle with a set of patches on its surface, mimicking different types of interaction points. With this model, Lu et al. [81] was able to simulate the formation of HbS fibers starting from pre-existing nucleates (see Figure 1d). In addition, the authors further demonstrated that the asymmetric structure of the HbS fiber mediates the stiffness of the fiber, implying that therapeutic agents that can modify the structure of fibers may inhibit the sickling of RBCs and thus ease the sickness.

**Figure 2:**
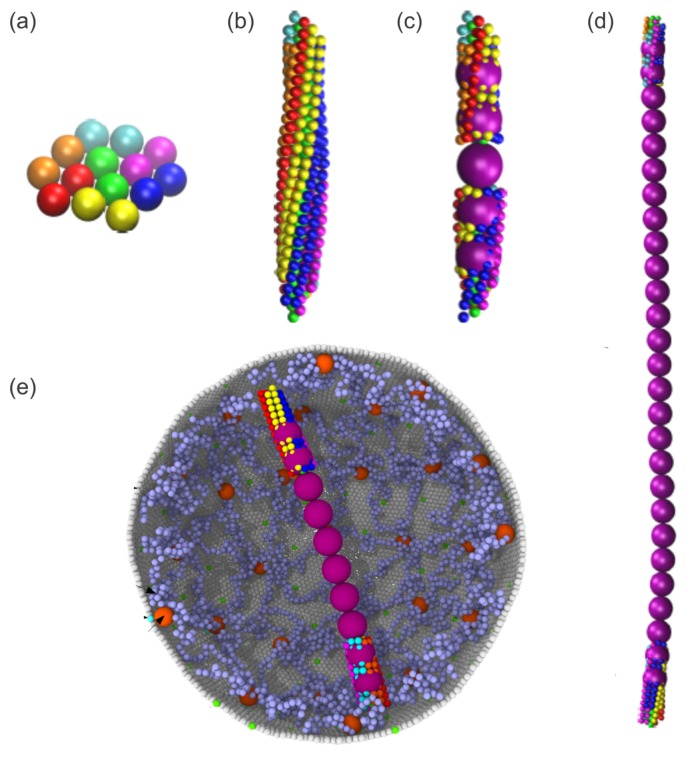
(**a**–**d**) Sequential snapshots of growth of a HbS fiber simulated by a hybrid HbS fiber model. Reproduced with permission from reference [85]. (**e**) Simulation of the interactions between a spherical RBC [61] and a HbS fiber using a hybrid fiber model [85].

Although this coarse-grained (CG) patchy particle model can capture the detailed structure of a HbS fiber, it is computationally prohibitive when simulating the integrated process of HbS fiber growth and the interaction between HbS fibers and RBC membrane. Therefore, multiple fiber models with higher levels of coarse-graining were developed [82,83,84], which are able to simulate the fiber–fiber interactions as well as the fiber-RBC interactions at a cellular scale. Recently, Lu et al. [85] extended the HbS patchy particle model by using an adaptive resolution scheme and developed a unique hybrid HbS fiber model. This new model couples the patchy particle model [85] with a coarser HbS fiber model [83]. As shown in Figure 2a–d, the patchy particle model is used to simulate the dynamic process of HbS polymerization, whereas the coarser fiber model preserves the mechanical properties of HbS fibers, thereby largely reducing the computational cost. Thus, this model provides the possibility to model the multi-scale process of RBC sickling with particle-based methods (see Figure 2e).

#### 2.1.1. Impaired RBC Deformability

**Figure 3:**
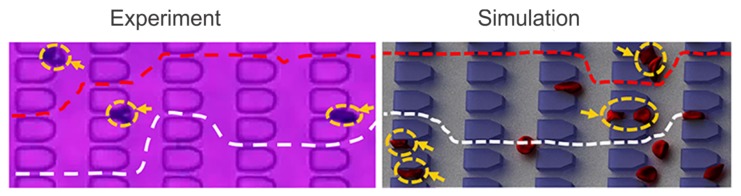
(**Left**) Microfluidics device showing individual SS-RBC passage through capillary-inspired microchannels under transient hypoxia. (**Right**) The corresponding simulations of SS-RBCs passage through capillary-like microchannels under transient hypoxia by dissipative particle dynamics. Reproduced with permission from reference [98].

SS-RBCs tend to acquire an abnormal, sickled shape when the intracellular HbS polymerizes as a result of low oxygen tension. Due to the presence of intracellular HbS fibers, SS-RBCs are more rigid than normal RBCs. Various experimental techniques have been implemented to measure the biomechanical properties of SS-RBCs, such as ektacytometry [86,87], RBC flickering analysis [88,89], atomic force microscopy (AFM) [34], microfluidic constriction channels [90,91,92] and micropipette aspiration [93], see review [94,95], which demonstrated convergent results on the increased stiffness of SS-RBCs. In particular, AFM measured a wider distribution of Young’s modulus from SS-RBCs [34] due to the variations in the hypoxic conditions and density of RBCs [96]. Du et al. [97] probed the deformability of SS-RBCs under transient hypoxia by using a high-throughput microfluidic device, where the kinetics of cell sickling and unsickling can be quantified (see Figure 3 (left)). Following this experimental study, Li et al. [98] performed computational simulations of SS-RBCs with a broad spectrum of morphological and biomechanical alterations passing through capillary-like microchannels under transient hypoxia (see Figure 3 (right)). The authors found that SS-RBCs exhibit significant heterogeneity when passing through capillary-like microchannels even within a particular density group. This study improved the understanding of possible RBC sickling during their capillary transition in vivo, and provided new insights into the underlying mechanism of vaso-occlusion events in SCD.

#### 2.1.2. Enhanced SS-RBC Adhesion and Vaso-Occlusion

Although the molecular basis of SCD is well understood, the underlying mechanisms of initiating and propagating vaso-occlusion events in SCD have not been fully elaborated. None of the clinically-measured SS-RBC markers, such as RBC indices, hemoglobin concentration, proportion of irreversibly sickled cells (ISCs), fetal hemoglobin level, reticulocyte count etc., can satisfactorily predict the severity of the patient’s clinical experience [99]. Hence, vaso-occlusion is believed to be multi-factorial in nature in that it involves not only cell sickling, but also the abnormal SS-RBC adhesion with other blood and immune cell populations, such as neutrophils, platelets, endothelial cells, and extracellular matrix proteins [15,100,101,102,103,104].

Key adhesion studies of Hebbel and Hoover et al. [99,105] directed scientific attention to the study of SS-RBCs in conjunction with the human vascular endothelium for the understudying of vaso-occlusive crisis events. These studies suggested that cell-cell interactions may be of pathophysiological importance in SCD and thus contribute to novel therapeutics for the disease. Hebbel et al. [99] used an in vitro assay in which endothelial cells were isolated from human umbilical cord veins and cultured in plastic flasks in confluence. Following that, washed SS-RBCs were statically incubated within the flasks for 30 min, and the adherence ratio was recorded. Their results showed that the adherence of SS-RBCs to endothelial cells is positively and significantly correlated with the individual’s clinical severity score (CSS), which is a number (between 1 and 11) assigned to the patients. This number collectively reflects the frequency of pain crisis, skin ulcerations, stroke events, and retinal and bone lesions. Hoover et al. [105] used a similar in vitro assay to measure the number of Cr-labeled SS-RBCs (ISC and non-ISC) versus healthy control RBCs adhered to endothelium cultured from calf aortas. They found that SS-RBCs exhibited a twofold increase in binding to endothelial cells compared to controls, confirming the results in reference [99]. These studies concluded that the propensity of SS-RBCs for adhesion is likely to initiate vaso-occlusion events by adherent SS-RBCs in post-capillary venules, which would either be sufficient to directly occlude the vessel at the site of adhesion, or would impede the flow upstream in the microcirculation, increasing the residence time of the already oxygen-depleted SS-RBCs in capillaries, hence allowing sufficient time for the cells to sickle.

**Figure 4:**
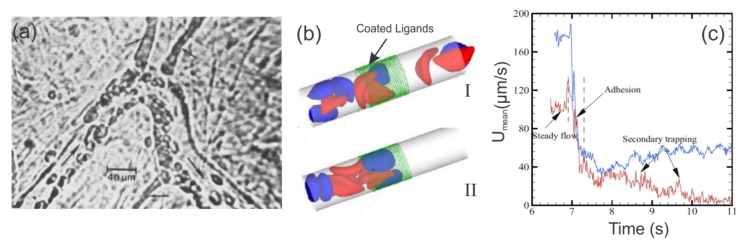
(**a**) SS-RBCs adhere to venular bending and to junctions of smaller diameter postcapillary venules (small arrows) in the isolated mesocecum observed by using intravital microscopy. The large arrows indicate the flow direction. Reproduced from reference[106] with permission. (**b**) Model of SS-RBCs flowing in capillaries. Reproduced from reference [110]. The green dotted region represents the ligands coated on the vessel wall. The blue cells represent the active group of SS-RBCs that exhibits adhesive interaction with the ligands on the wall. The red cells represent the non-active group of cells. (**b**. (I)) A snapshot of SS-RBCs in a non-occlusion state, showing the active and non-active cell groups flowing unobstructed within the vessel. (**b**.(II)) A snapshot of SS-RBCs in an occlusion state. The active (blue) cells adhere to the wall by interacting with the ligands. (**c**) Mean velocity as a function of time. The red and blue curves correspond to different pressure drops (red: 8.3 × $10^{4}$ Pa/m and blue: 1.35 × $10^{5}$ Pa/m). Prior to adhesion (t < 6.9 s), a steady flow state is reached within the vessel with average velocities of ∼115 μm/s (red), and ∼180 μm/s (blue). When the adhesive forces are turned on (6.9 s < t < 7.3 s), the average velocity exhibits a sharp decrease to ∼40 μm/s, and blood flow exhibits a transition from a steady flow state to a partially (blue) or even completely (red) occluded state. The velocity of the blood flow of the red curve (smaller pressure drop) decreases to ∼10 μm/s, representing the secondary entrapment of the non-active cells and the fully occluded state. Reproduced from reference [110].

In later studies, Kaul et al. [106,107,108] further unraveled the type of adhesion molecules involved in the SS-RBC/endothelium interactions. They infused human SS-RBCs into rats whose endotheliums had been activated by platelet-activating factor (PAF) and used intravital microscopy to monitor the blood flow and adhesion of SS-RBCs to vessel endothelium (Figure 4a). In the absence of antiadhesive agents, i.e., the monoclonal antibodies 7E3 (blocks integrin avb3 and glycoprotein IIb/IIIa) and LM609 (selective blocker of integrin avb3), they showed enhanced adhesion in postcapillary venules accompanied by occasional obstruction. They also showed that preferential adhesion of low-density SS-RBCs and reticulocytes in immediate post capillary venules leads to trapping of the older, more dense SS-RBCs, resulting in frequent blockage of small diameter venules. This finding was later confirmed by a microfluidic study of SS-RBCs flowing around an acute corner of a triangular pillar [109]. In contrast, when the animals were treated with the monoclonal antibodies (7E3 or LM609), SS-RBCs did not adhere to the endothelium and the flow was unobstructed [107,108].

Based on experimental observations, extensive computational studies of SS-RBC hemodynamic and rheological characteristics have been made in the past ten years. Lei and Karniadakis [110] coupled Dissipative Particle Dynamics (DPD) based RBC model with adhesive dynamics model to simulate the flow of SS-RBCs in a small tube under constant shear flow, accounting for the abnormal morphology and altered membrane mechanics of SS-RBCs under hypoxia. Their simulations validated the dependence of SS-RBC rheology on cell shape, as previously reported in reference [111]. Furthermore, as shown in Figure 4b,c, they validated the two-step SS-RBCs/endothelium occlusion model [14,19,106], which states that vaso-occlusion is initiated with the adhesion of highly-deformable SS-RBCs to the endothelium of the vessel walls and is propagated by the more rigid SS-RBCs that start to accumulate behind the adhesion site that will eventually lead to an occluded vessel. In Lei et al.’s simulations [110], the adhesive cells (blue) attached on the vessel walls resulted in elevation of the flow resistance. These adhesive cells can further entrap non-adhesive, but less-deformable cells which leads to secondary elevation of flow resistance or even full occlusion of the vessel.

**Figure 5:**
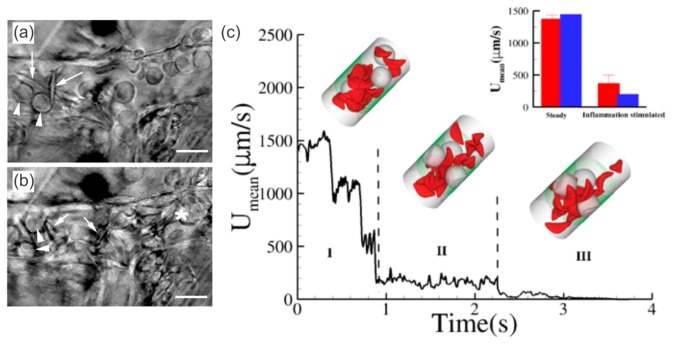
Still frames of intravital microscopy showing (**a**) interactions between SS-RBCs (arrows) and adherent leukocytes (arrow-heads) in the venules of sickle mice and subsequent (**b**) venular occlusion (white asterisk). Reproduced from reference [112]. (**c**) In silico studies of vessel occlusion induced by inflammation-stimulated leukocytes. Instantaneous mean velocity of the blood flow in a vessel of diameter of D = 20.4 μm and Hct = 13% encompassing three leukocytes. (Insets) The green dotted region represents the coated ligands, mimicking the inflammation region of the vessel. Snapshots represent blood flow states as follows: (I) initial stage of inflammatory response and free motion of the blood flow; (II) moderate RBC-leukocyte interactions and blood flow slowdown; (III) late stage of the inflammatory response, where the RBC-leukocyte interaction is further intensified, leading to entrapment of multiple SS-RBCs on the adherent leukocytes and consequent vessel occlusion. (Inset plot) Side-by-side comparison of experiments versus simulations. The blue bars represent the blood flow velocity of the present study and the red bars represent the experimental results in reference [100], where measurements were taken on 23–41 venules with average diameters of 20.9 ± 1.3 μm and 24.9 ± 1.8 μm before and after inflammation stimulation. Reproduced from Lei et al. [110].

More recently, in vivo studies revealed the role of adherent leukocytes in sickle cell vaso-occlusion (see Figure 5a,b), beyond the two-step endothelium-RBC adhesion/occlusion model. Frenette et al. [100,112] employed intravital microscopy in transgenic humanized sickle mice (mice that exclusively express human HbβS) and showed that leukocytes play an important role in occlusion via enhanced endothelium-leukocyte adhesion and leukocyte-RBC binding in inflamed venules [15,102]. Frenette et al. [113] also showed that inflammation (i.e., elevated platelet and leukocyte counts) is associated with sickle vaso-occlusion. Hence, they chose the pro-inflammatory cytokine Tumor Necrosis Factor-α (TNF-α), which induces P- and E-selecting mediated leukocyte rolling, to induce leukocyte adhesion [113]. They found that sickle mice pretreated with TNF-α cytokine exhibited leukocyte adhesion on the vessel walls and died soon or after the cremasteric preparation. In contrast, control mice and sickle mice that did not receive TNF-α treatment did not exhibit leukocyte adhesion and survived.

As shown in Figure 5c, Lei and Karniadakis [110] simulated leukocyte/RBC vaso-occlusion in small vessels via a DPD adhesion dynamics model. They used a stochastic association/dissociation model to represent the formation and rupture of bonds between blood cells as well as between cells and vessel walls over time. This model was validated by comparing their simulation results (inset plot of Figure 5c) against the experimental data in reference [100]. The authors further quantified the influence of adherent leukocytes, which may arrest SS-RBCs and cause partial or complete vessel occlusion.

The latest sickle cell vaso-occlusion model is believed to be multi-cellular and multi-step and involves adhesive interactions amongst SS-RBCs, neutrophils, and endothelial cells as follows: activated endothelium attracts the integrin-mediated adhesion of neutrophils. Subsequently, neutrophils arrest circulating SS-RBCs mediated by CD11b/CD18 (Mac-1) integrin [102]. In this vaso-occlusion model, the aged neutrophils play an important role due to their enhanced Mac-1 surface expression [102]. Furthermore, recent in vivo and in vitro studies established the role of platelets in the vaso-occlusion cascade [104,114,115]. Bennewitz et al. [104] monitored the interactions between platelets and arrested neutrophils using quantitative microfluidic fluorescence microscopy, through which the authors found enhanced neutrophil-platelet aggregation in SCD human whole blood compared to African American healthy controls.

**Figure 6:**
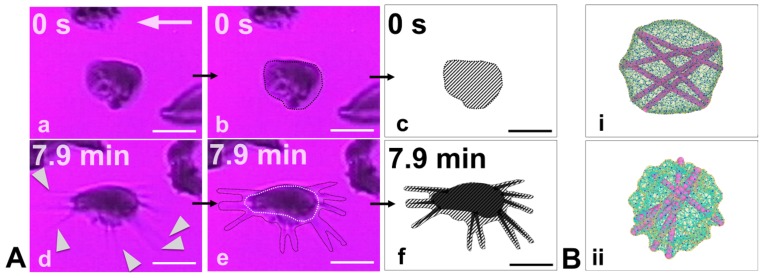
(**A**) Experimental results of simultaneous adhesion and polymerization in sickle reticulocytes under hypoxia and shear flow on a fibronectin-coated microchannel wall. (**a**) (t = 0) The cell adheres on the surface. (**d**) (t = 7.9 min) During cell adhesion, there is significant protrusion of polymerized HbS fibers (white pointers) outwards of the bulk of the cell. (**b**,**e**) Outline of the contours of the initial and final (including the HbS protrusions) snapshots of the adherent sickle reticulocyte. (**c**,**f**) Hatched sketches of the cell-wall contact area. The hatched area roughly represents the contact area of the cell’s lipid bilayer. The hatched area in snapshot (**c**) is approximately two times larger than the hatched area in snapshot (**f**). The white arrows denote the flow direction. Scale bar: 5 μm. From Papageorgiou et al. [116] with permission. (**B**) Simulation results of HbS polymerization within a mature sickle cell (**i**) versus a sickle reticulocyte (**ii**). The excess membrane of the sickle reticulocyte in (**ii**) (that has not been shed in the circulation yet through vesiculation) allows the polymerized HbS fiber projections to continue to grow outwards of the cell bulk while the membrane is simultaneously encompassing the fibers, i.e., confirming the experimental observations in (**A**).

Recently, Papageorgiou et al. [116] showed the unique adhesion dynamics of sickle reticulocytes (under hypoxia) (see Figure 6a–f), the HbS fiber projections can extensively grow outward of the cell boundary, creating multiple adhesion sites. They also showed that not only in reticulocytes, but also in young erythrocytes, adhesion and HbS polymerization can work synergistically to increase the number of adhesion binding sites while the cell is adhered on the surface within minutes. The aforementioned mechanisms may prove to be factors in initiating or promoting SCD vaso-occlusion. Furthermore, Papageorgiou et al. [116] suggested a connection between polymerization, adhesion, and SS-RBC maturation, which resulted in the following descending order of the degree of adhesion susceptibility under hypoxia: sickle reticulocytes in the circulation ⇒ mature SS-RBCs with low density and high deformability ⇒ mature SS-RBCs with high density and low deformability ⇒ irreversibly-sickled cells.

## 3. Hereditary Spherocytosis

### 3.1. RBC Vesiculation

**Figure 7:**
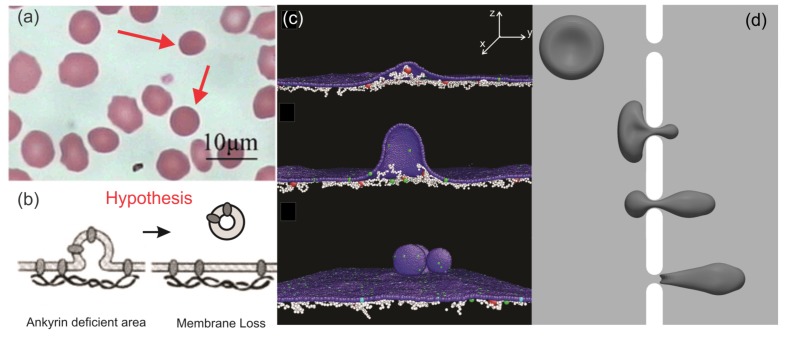
(**a**) Optical microscopy images of peripheral blood smears of HS patients after splenectomy. Reproduced with permission from reference [119]. (**b**) Hypothesis for RBC membrane loss in HS proposed in reference [120]. (**c**) Simulations of membrane vesiculation of defective RBC membranes in HS using the coarse-grained molecular dynamics (CGMD) method. Reproduced from reference [121] with permission. (**d**) Simulations of a RBC passing through a narrow slit using the finite element method. Reproduced from reference [122] with permission.

HS, another type of RBC disorder, is induced by mutations of RBC membrane proteins, such as ankyrin, protein 4.2, band-3, spectrin, and so on, which are responsible for the linkages between the lipid bilayer and the skeleton of RBCs [21]. These protein defects weaken the vertical integrity between the two layers of the RBC membrane, causing loss of cell surface area, which is thought to occur through vesiculation [21]. Due to the reduced surface area, RBCs in HS transform gradually from biconcave shapes to spherical shapes [8], as shown in Figure 7a. Meanwhile, the deformability of RBCs decreases because of the reduced surface area to volume ratio [117], leading to premature removal by the spleen [118]. Although the molecular basis of HS has been unveiled, the mechanics of the membrane loss are not fully understood. Two prevailing hypotheses [21] are as follows: (i) in spectrin-deficient RBCs, lipids that are unsupportive by the cytoskeleton can bud off and form vesicles, resulting in membrane loss, and (ii) in band-3 or protein 4.2-deficient RBCs, the connections between the spectrin filaments and lipid bilayer are reduced, causing compromised cohesion between the two layers of membrane and consequent vesiculation (see Figure 7b). In addition, it is broadly considered that RBCs in HS shed vesicles predominantly during their sojourn in the circulation [21]. However, no clinical evidence can be provided to support these hypotheses as the vesiculation of RBCs cannot be directly observed in vivo.

Motivated by these prior clinical observations and hypothesis, computational modeling was employed to investigate the underlying mechanism of surface area loss of RBCs in HS. Spangler et al. [123] modeled the membrane budding of spherical RBCs by the coarse-grained molecular dynamics (CGMD) method. They demonstrated that membrane budding can be triggered by localized disruption of the cytoskeleton, thereby confirming the hypothesis raised in reference [120]. Along this line, Li et al. applied CGMD RBC membrane models [60,124] to simulate vesiculation of the HS RBC membrane considering the lateral compression on the membrane, as shown in Figure 7c. The authors discovered that as the vertical connectivity between the lipid bilayer and membrane skeleton reduced, the surface area loss was exacerbated [121]. These results indicate that numerical modeling can facilitate the laboratory studies on defining the connection between genetic basis of the RBC disorders with their clinical manifestations. Furthermore, the authors reported that when membrane domains with spontaneous curvatures are considered, they tend to release vesicles that originate from curved membrane domains. Chang et al. [117] investigated the single cell dynamics of HS RBCs by coupling a CGMD RBC model [60] with a dissipative particle dynamics RBC model [59], through which they found that the weakened bilayer-cytoskeleton interactions delay the transient shape relaxation of HS RBCs. In particular, HS RBCs lose their ability to recover the normal biconcave shape in successive loading cycles of stretching and relaxation, leading to irreversible deformations. Zhu et al. [122] simulated RBC passage through a narrow slit using the finite element method to mimic the traversal of inter-endothelium slits in the spleen (see Figure 7d). Based on the simulation results, the authors predicted that vesiculation from RBCs in HS may occur during their splenic transition where RBCs undergo drastic deformations. This finding provides a possible mechanism of RBC vesiculation in HS and thus explains the presence of spherical RBCs in the blood smear of HS patients. Recently, Li et al. [125] demonstrated that HS reticulocytes are more prone to shedding surface area than mature RBCs by using CGMD simulations. This result confirms a previous clinical finding that RBCs in HS start shedding membrane more aggressively at the reticulocyte stage [126].

### 3.2. Membrane Protein Diffusion

In addition to maintaining the elasticity and stability of RBCs, the cytoskeleton functions as a physical barrier hindering the lateral diffusive motion of band-3 proteins [127,128,129,130,131]. Tomishige et al. [128] implemented the single particle tracking technique to study the diffusive motion of band-3 proteins and their interaction with the membrane cytoskeleton. In their experiments, gold particles of 40 nm conjugated with Fab fragments of anti-band 3 antibodies were attached to band-3 proteins on human RBC ghosts. The movement of gold particles was recorded by a contrast enhanced bright-field optical microscopy. The authors discovered that approximately one-third of the band-3 proteins are connected to the cytoskeleton and thus exhibit highly restricted diffusive motion (immobile band-3). The remaining two-thirds of the band-3 proteins diffuse freely (mobile band-3) within small membrane compartments formed by the spectrin filaments on a short time scale ( less than 10 ms). On larger time scales, these band-3 proteins undergo a series of confined diffusive motions due to the hindrance of the cytoplasmic parts of band-3 proteins by the spectrin filaments. This finding was subsequently confirmed by the fact that after the band-3 protein is cleaved (removal of the cytoplasmic part), the diffusivity of band-3 is significantly enhanced [130]. Occasionally, these confined band-3 proteins hop to the neighboring compartments, leading to normal diffusive motion on a large time scale. Similar diffusive motions were observed in fluorescence photobleaching recovery experiments [127,132,133,134,135], where freshly washed RBCs were incubated with fluorescent molecules such that fluorescent molecules were bound to band-3 proteins. A laser beam was used to provide intense photobleaching pulses on a spot on the labeled RBCs. After a photobleaching pulse, unbleached molecules attached to the band-3 proteins diffuse to the bleached area, through which the diffusive motion of band-3 in healthy and diseased RBC membranes can be quantified. Based on these experimental observations, two types of lateral diffusive motions were summarized for mobile band-3 proteins [135]: (i) the microscopic diffusion describes the diffusive motion of band-3 proteins within spectrin network compartments and (ii) the macroscopic diffusion accounts for the long-term diffusive motion of band-3 proteins, involving hindrance by spectrin filaments and occasional hop motions.

The diffusive motion of band-3 proteins in RBC membrane has been studied extensively as it can be considered as a biomarker for the severity of blood disorders [127,136,137] or for determining the maturation stage of RBCs in erythropoiesis [138]. Prior studies pointed out that the diffusivity of band-3 proteins is increased in HS and hereditary elliptocytosis (HE) [132,134,136]. Furthermore, the levels of abnormal diffusive motion of band-3 proteins may be correlated with the severity of RBC disorders. In HS, mutations of RBC membrane proteins damage the vertical cohesion of the cell membrane. In HE, protein defects at the spectrin dimer-dimer connection sites or at the actin-spectrin junctions of the cytoskeleton, impair the cohesion of the cytoskeleton. Both types of alterations can modify the lateral diffusion of the mobile band-3 proteins.

**Figure 8:**
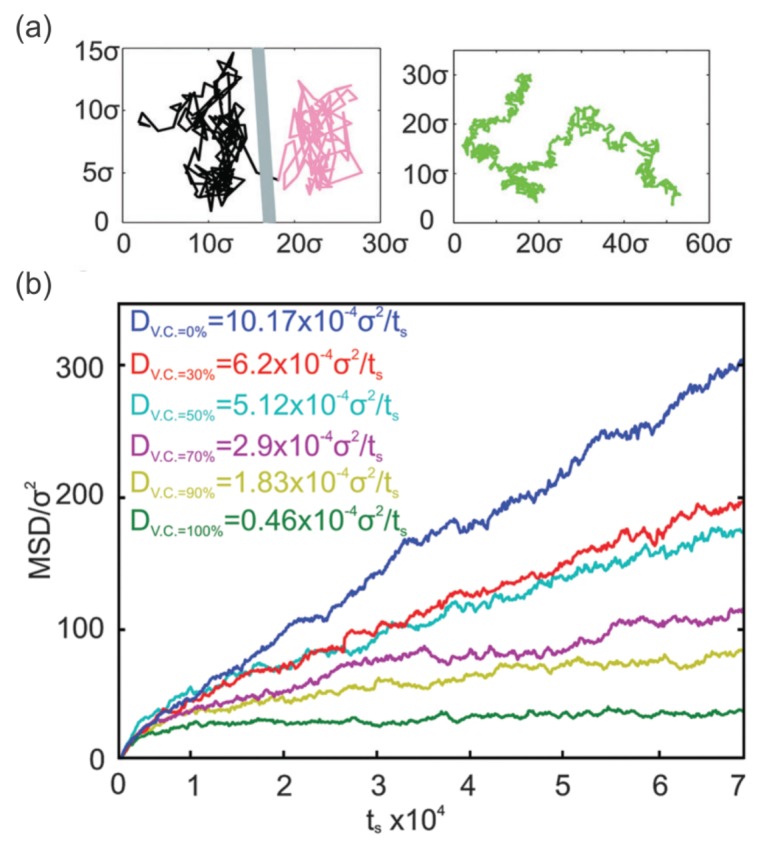
(**a**) Trajectory of a mobile band-3 protein in an RBC membrane undergoing hop diffusion (**left**). Trajectory of a mobile band-3 protein in a lipid bilayer undergoing normal diffusion (**right**) obtained from CGMD simulations [140]. (**b**) Mean square displacement (MSD) of mobile band-3 proteins with varying vertical connectivities between band-3 proteins and spectrin filaments. Reproduced from reference [140] with permission.

Extensive experiments have been conducted to understand the diffusive motion of band-3 proteins in healthy and diseased RBC membranes, but quantitative correlations between the diffusivity of band-3 proteins and the severity of diseases is still missing due to limitations in the experimental technique. Numerical modeling, on the other hand, is able to fill this gap. Saxton [129,139] performed Monte Carlo simulations to investigate the dependence of the band-3 protein diffusivity on the cytoskeleton integrity and demonstrated that the band-3 diffusion coefficients dropped significantly with increased cytoskeleton connectivity. However, membrane fluctuation was not considered in these simulations. Auth et al. [130] proposed an analytical model to investigate the band-3 diffusive motion in the membranes of healthy RBCs and in the membranes with reduced vertical connectivities, mimicking defects in ankyrin proteins. Their results demonstrated that the defects in ankyrin proteins caused enhanced band-3 protein diffusion, consistent with the clinical studies in reference [136]. Li et al. [140] studied the normal band-3 protein diffusion in the lipid bilayer and abnormal diffusion in the RBC membrane by employing a CGMD RBC membrane model, as illustrated in Figure 8a. They quantified the correlations between band-3 protein diffusion coefficients and the level of protein deficiency in HS RBCs (see Figure 8b). These findings provide a clarification on the connection between the severity of the molecular deficiencies and the diffusivity of band-3 proteins in HS RBCs. Furthermore, the authors found that the association between the cytoskeleton and the lipid bilayer, other than at the band-3 binding sites, hinders the motion of band-3 proteins in healthy RBC membranes and in the RBC membrane with HS. They also estimated that the scale of the effective association force between the spectrin filaments and lipid bilayer, which cannot be measured with currently available experimental approaches, is at least 20 times smaller that of the spectrin-band 3 binding sites [140].

## 4. Diabetes Mellitus

### 4.1. Mechanics of Diabetic RBCs

DM, recognized as the world’s fastest growing chronic disease, is a metabolic dysfunction of the persistent hyperglycemia caused by either insulin insufficiency or insulin resistance at many body cells [141]. T2DM is the most common form of diabetes. People with T2DM have a higher risk of having cardiovascular diseases and stroke which can cause severe heart damage or mortality [142]. Hematological abnormalities that emerge in diabetic patients play key roles in the pathogenesis and progression of life-threatening diabetic complications [143,144,145]. One of the hemorheological determinants is the impaired deformability of RBCs involved in T2DM. Using micropipette aspiration, McMillan et al. [28] observed an elevated pressure gradient for a standard oscillatory movement of diabetic RBCs compared with non-diabetic subjects and concluded that RBC deformability is reduced in DM. It is likely that a rise of intracellular viscosity associated with increased glycated hemoglobin (HbA1c) have contributed to the resistance to deformation. RBC deformability is a critical factor for blood rheology in microcirculation. Tsukada et al. [146] measured erythrocyte deformability by estimating the deformation index (DI) of flowing RBCs in the transparent crystal microchannels. The DI values of diabetic RBCs were shown to be significantly smaller than those of healthy controls. Agrawal et al. [29] showed decreased DIs of RBCs from T2DM patients compared with RBCs from healthy people by using a dual optical tweezer stretching technique. They also showed a great decrease in the DIs of RBCs from patients with diabetic retinopathy. Moreover, numerous AFM studies have directly pointed out an evident stiffness increase in diabetic RBCs with an increase in the average Young’s modulus [32,147,148]. This could be due to the glycosylation of the RBC membrane during hyperglycemia which, in turn, leads to alterations in the membrane lipid-protein interactions associated with the variations in membrane viscoelastic properties.

**Figure 9:**
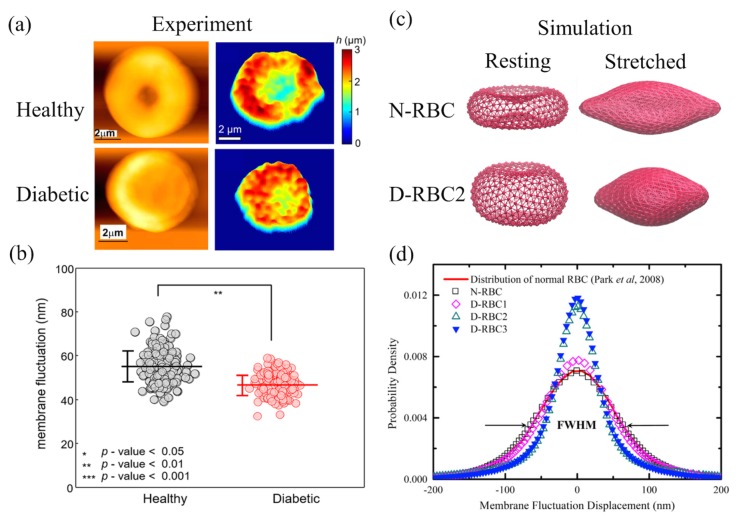
(**a**) Morphological characteristics of a representative healthy red blood cell (RBC) and a diabetic RBC depicted in the AFM images (**left**). Reproduced from reference [150] with permission. Two-dimensional topographic height maps (**right**). Reproduced from reference [151] with permission. (**b**) Scatter plot of retrieved membrane fluctuations from RBC data in healthy and diabetic groups where the horizontal lines are the mean values of membrane fluctuations and the vertical error bars are the sample standard deviations. Reproduced from reference [151] with permission. (**c**) Coarse-grained models of RBCs in healthy (N-RBC) and in diabetic (D-RBC3) patients with their resting forms (**left**) and the stretched states under external tensile force 100 pN (**right**). Reproduced from reference [152] with permission. (**d**) Membrane fluctuation distributions of different RBC models [152]. N-RBC is a representative model for normal RBCs, while D-RBC1, D-RBC2, and D-RBC3 are three potential models for diabetes mellitus (DM) RBCs. The simulation results of N-RBC is compared with the experimental data [153] drawn in red line and FWHM is the full-width half-maximum. Reproduced from reference [152] with permission.

A healthy RBC has a distinctive biconcave shape endowing itself a large S/V ratio and remarkable deformability. RBCs in T2DM might become more swollen or irregular because of the possible metabolic disturbances [29,149,150]. Jin et al. [150] revealed a visible shape change from the typical biconcave shape to a near-oblate shape with a reduced S/V ratio in the T2DM RBCs. As shown in the AFM images in Figure 9a (left), a deeper dimple region is present in healthy RBCs, while a shallow dimple or no dimple occurs in diabetic RBCs. Similar results were presented by Lee et al. [151] via common-path diffraction optical tomography (cDOT). The 2D membrane height maps of RBCs from healthy controls and from diabetic patients in Figure 9a (right) imply a loss of the center dimple region for their representative diabetic RBCs. Lee et al. [151] also estimated the mean membrane fluctuation by spatially averaging the 2D membrane fluctuation map of individual RBCs (see Figure 9b). They found a significant decrease in the mean membrane fluctuation of diabetic RBCs compared to healthy RBCs, which indicates an impaired deformability of diabetic RBCs. In addition, they observed a negative correlation between RBC membrane fluctuation and HbA1c, suggesting that the membrane fluctuation decreases as the HbA1c level increases.

Chang et al. [152] recently developed and proposed three potential T2DM RBC models by considering different hallmarks observed in the pathophysiology of T2DM RBCs [150,154,155]. In the first T2DM model (D-RBC1), the RBCs have a typical biconcave shape as in normal RBCs (N-RBC) but they have an increased shear modulus. The second and third T2DM models (D-RBC2 and D-RBC3) have near-oblate shapes and an increase in membrane stiffness. In particular, D-RBC3 has an additional trait of enhanced membrane viscosity. As shown in Figure 9c, a normal RBC model (N-RBC) is a biconcave shape in its resting form, while in the second T2DM RBC model (D-RBC2), it adopts a near-oblate shape. The stretched states of N-RBC and D-RBC2 under an external tensile force of 100 pN are also presented in Figure 9c. It is obvious to see the reduced cell stretching response of D-RBC2 against N-RBC at the same tensile force. Chang et al. [152] also found a diminished membrane fluctuation reflected in the narrower fluctuation distribution in Figure 9d of the T2DM RBCs compared to the normal RBCs, which is qualitatively consistent with experimental work (Figure 9c) by Lee et al. [151]. As for T2DM RBCs with the abnormal near-oblate shape (D-RBC2 and D-RBC3), much narrower fluctuation distributions are presented (see Figure 9c). These computational results indicate that both the membrane shear modulus and shape alteration are crucial in RBC stretching behaviors and membrane fluctuations.

### 4.2. Biorheology of Diabetic Blood

**Figure 10:**
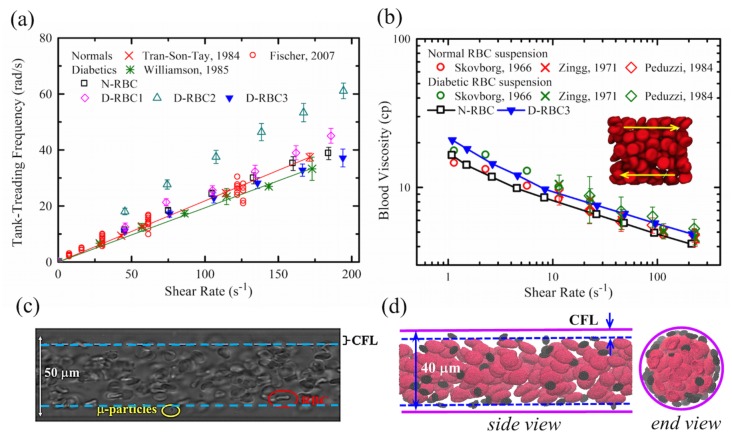
(**a**) Normal and T2DM RBC tank-treading frequency as a function of the shear rate. Simulation results [152] compared with experimental data by Fischer (red circle) [156], by Tran-Son-Tay et al. (red cross) [158], and Williamson et al. (green star) [155]. Linear fits for the experimental data of normal RBCs (red line) and diabetic RBCs (green line). Reproduced from reference [152] with permission. (**b**) Blood viscosity of normal and T2DM RBC suspension as a function of shear rate at a hematocrit of 45%. Simulation results (squares and triangles) [152] compared with experimental data (circles [144], crosses [161], and diamonds [162]). The red and green symbols are for the normal and diabetic RBC suspensions, respectively. Reproduced from reference [152] with permission. (**c**) Typical particle/platelet margination observed in blood flow from an in vitro study [163]. Reproduced from reference[163] with permission. (**d**) A DPD simulation of RBCs and platelets flowing in a circular channel showing an apparent cell-free layer (CFL).

Deformable RBCs can exhibit tank-treading (TT) motion, characterized by a steady orientation and membrane circulation, when they are subjected to high levels of shear stress [156,157]. Tran-Son-Tay et al. [158] and Williamson et al. [155] investigated the TT frequency (*f*), i.e., the number of TT cycles per second, of RBCs in normal and diabetic conditions. They obtained a linear relationship between *f* and the shear rate $\dot{\gamma }$ and found a slight decrease in TT frequency of diabetic RBCs compared to normal RBCs. Following these experimental studies, Chang et al. [152] performed a systematical TT motion of RBCs using normal and T2DM RBC models in linear shear flow, as shown in Figure 10a. They first validated their normal RBC model with the corresponding TT frequency measured in experiments. Then, they demonstrated positive correlations between *f* and $\dot{\gamma }$ for all three T2DM RBC models. However, among all T2DM models, only D-RBC3 was shown to fit well with the measured TT frequency of diabetic RBCs by Williamson et al. [155], suggesting that D-RBC3 with explicit consideration of increased effective membrane viscosity leads to a more accurate model of the impaired RBC dynamics in T2DM. It is noted that a recent work by Lanotte et al. [159] found rich dynamic morphologies of RBCs with blood shear thinning by using microfluidic rheometry and numerical approaches. They showed unexpected RBC dynamics characterized by rotating polylobed shapes instead of a steady TT motion at a relatively high viscosity contrast. Their studies suggested that any pathological changes in the inner-to-outer viscosity ratio and RBC deformability alter the RBC dynamics and the onset of shape transitions.

RBC deformability is of importance to determine the blood flow resistance in the microcirculation. Abnormally high blood viscosity has been found in patients with DM which is related to the development of diabetic microangiopathy [29,30]. Experimental studies focused on the blood viscosity of non-diabetic and diabetic blood have been comprehensively reviewed by Cho et al. [160] The T2DM model of Chang et al. [152] shows good predictions for the increased blood viscosity in T2DM and good agreements with experimental data (Figure 10b). Although their T2DM models currently do not consider the cell-cell aggregation interactions and thus fail to model the rouleaux structures formed at low shear rates, the models with explicit description of the T2DM RBC structure and membrane properties still provide useful insights into the correlations between the reduced cell deformability and increased blood viscosity in T2DM.

Another important hemorheological determinant in the development of cardiovascular disease is the thrombotic abnormality for diabetic patients [164]. It includes elevated levels of numerous coagulation factors such as tissue factors, thrombin, and fibrinogen, a reduction in clot lysis, and platelet hyperreactivity [165,166]. There is growing evidence that diabetic patients have a high mean platelet volume (MPV) and this size alteration leads to an increased platelet adhesion, activation, and aggregation, and therefore facilitates thrombosis [167,168]. Platelet dynamic transport to an injured site is the preliminary stage for the formation of platelet-subendothelium and platelet-platelet bindings. In a shear flow, rigid particles or non-deformable cells, such as leukocytes and platelets, can easily diffuse to the near-wall region in the bloodstream, known as “margination” [169]. Figure 10c,d the illustrate typical margination phenomena occurring in an in vitro study of micro-particle delivery [163] and a simulation study of platelet transport in the RBC suspensions, respectively. Because of the high deformability, RBCs tend to drift away from a confining wall and create a cell-free layer (CFL) region, while micro-particles and platelets are prone to be found in this layer [169,170,171,172,173]. Diabetic RBCs with impaired deformability and diabetic platelets with higher MPV could have influences on platelet margination behavior, which further exacerbates thrombus formation. Chesnutt and Han applied a discrete element method to study the effects of MPV, mean platelet density (MPD), and vessel tortuosity on platelet activation and thrombus formation in arterioles [174]. They found that an increase in MPV results in a larger number of activated platelets, while MPD and the level of turtuosity are less sensitive to platelet activation.

## 5. Future Prospectus

Although integration of experimental approaches and computational modeling have improved our understanding on the pathogenesis and pathophysiology of various blood diseases, there are still many questions that require further investigation. For example, the interactions between blood cells, such as red cells, white cells, platelets as well as the interactions between these blood cells and blood vessel endothelium under various flow conditions have not been well studied. In particular, there is a lack of experimental studies that quantify the changes of cell-cell interactions under pathological conditions, such as SCD, HS, and diabetes [175]. As a result, most numerical studies have to employ simplified interaction potentials to represent the cell-cell interactions. For example, Fedosov et al. [176] simulated the RBC aggregation and the formation of rouleaux structures by applying a Morse potential between RBCs to mimic adhesive interactions. The adhesive forces can be tuned by changing the parameters in the Morse potential. Lei and Karniadakis [110] simulated the interactions between the adherent leukocytes and sickled RBCs following the same strategy. It is noted that the model parameters in these studies were calibrated based on the static force measurements and thus may not be able to accurately capture the dynamic behaviors of blood cells flowing in the vessels. Therefore, future experimental studies on the interactions between diseased blood cells under dynamic flow conditions should be encouraged to improve accuracy and capability of the computational models in making predictions.

In addition, the integration of experimental approaches with computational modeling can be further extended to study biomechanics of RBCs in metabolic disorders induced by enzyme defects [177]. Mature RBCs are nucleus-free cells and there is no gene replication, transcription, or translation occurring inside RBCs. The major metabolic pathways in human RBCs are the Embden-Meyerhof pathway and the pentose phosphate pathway, which are responsible for maintaining the shape and deformability of RBCs as well as their function in carrying oxygen [177,178]. Due to the limited metabolic activities, RBCs have long been used to elaborate the fundamental metabolic pathways. A number of metabolic models of human RBCs have been developed in the past two decades [178,179,180,181], in which the RBC metabolism is described with a group of ordinary differential equations with different levels of detail, depending on the focus of the model. However, there are limited studies on metabolism-related pathological alterations of the biomechanics and morphology of RBCs. In addition, the metabolism pathways were mostly studied under static conditions without considering the environmental effects, such as the effects of the blood flow and RBC deformations. These environment effects can alter the transport of glucose into RBCs and the subsequent adenosine triphosphate (ATP) production which, in turn, change the morphology and dynamics of RBCs, as observed in reference [182]. Therefore, the development of a comprehensive human RBC model by integrating a particle-based RBC dynamic model with metabolic models is required to describe the mechanics and morphology of RBCs in metabolic disorders. Metabolic models with different levels of detail can be incorporated based on the focus of the specific studies. This type of comprehensive RBC model will be able to elucidate the complex relationships between the environmental effects, genetic basis of enzyme disorders, and clinical or laboratory manifestations of RBCs.

## Figures and Tables

**Table 1:** Summary of recent computational and experimental methods for studying blood flow.

Category	Method	Description	Reference(s)
Computational	Finite Element Method (FEM) and its variants	Suitable for boundary with complex geometry or irregular morphology. The space-time FEM was developed for moving-mesh methods. The Spectral/hp Element Method achieves high-accuracy but sometimes requires intense computation.	FEM [183,184,185,186,187]; space-time [188,189]; Spectral/hp [190,191]
	Finite Volume Method (FVM)	Easy application for unstructured mesh, which is often used for irregular boundary geometry.	[192,193,194,195]; ANSYS Fluent [196,197,198].
	Immersed Boundary Method (IBM)	A versatile method that easily couples with any existing solvers, like FEM, FVM, and the Lattice Boltzmann Method (LBM).	[66,199,200]; couple LBM [67,68] and FEM [201].
	Arbitrary Lagrangian-Eulerian Method (ALE)	Frequently used for large vessel flow and sometimes coupled with FEM.	[202,203]; couple FEM [187,204,205,206].
	Dissipative Particle Dynamics (DPD)	Particle-based coarse-grained method with artificial viscosity and dissipativity to recover Navier-Stokes equations.	[55,58,110,207,208,209].
	Boundary Element Method (BEM)	The most useful method for infinite flow problems, but limited to the low Reynolds number condition (i.e., Stokes flow).	[65,210,211,212,213,214,215,216,217].
Experimental	Microchips manufactured by modern material	Deformable materials such as Polydimethylsiloxane-made tubes mimic gas-permeable vessels or other organ tissues. Flexible micro-posts in flow were used to measure shear force of cells. Polymer brushes approximate glycocalyx linings.	[218,219,220,221,222,223,224,225].
	Geometry designs of flow system in vitro	Bifurcated or tortuous channels mimic complicated vascular networks. The tapered channel introduces continuously varying shear rates or nutrients. A sudden contracted channel was used to mimic a stenosed arteriole.	[226,227,228,229,230,231,232,233].
	Up-to-date measuring technology	RBCs are divided into different density groups when subjected to magnetic or electrical forces such that the deformability of cells in different groups could be measured. Particle imaging velocimetry (PIV) is introduced to profile the surfaces of blood vessels and measure flow speed.	electrical [234,235]; magnetic [236,237]; PIV [238,239,240].
